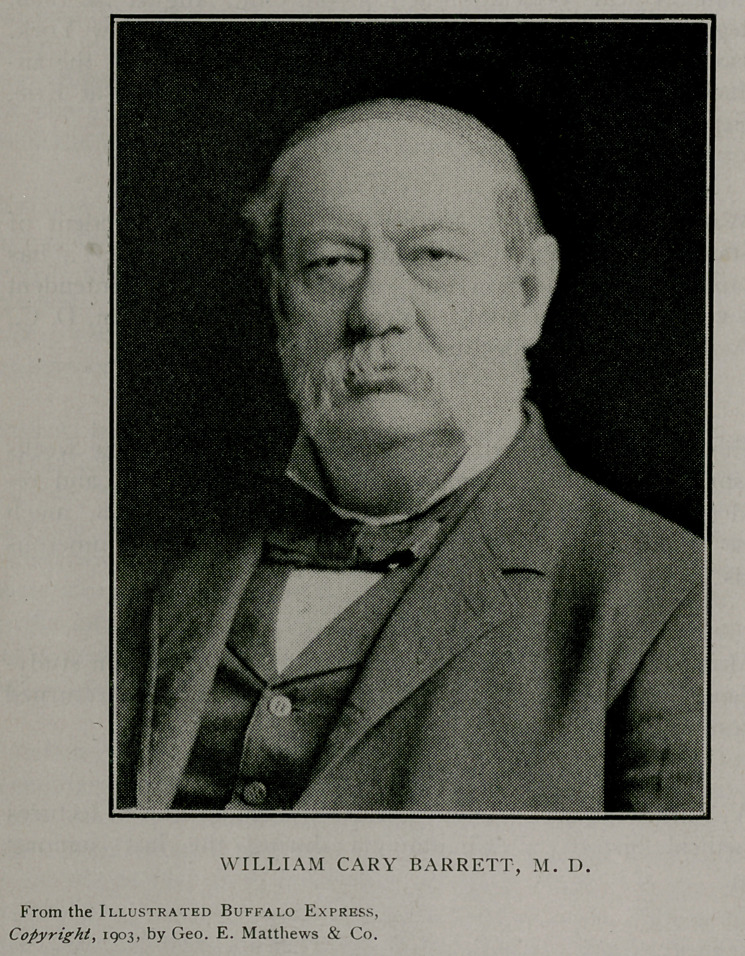# Dr. William Cary Barrett

**Published:** 1903-10

**Authors:** 


### OBITUARY.

Dr. William Cary Barrett, D.D.S., dean of the dental department of the University of Buffalo, died at Nauheim, in Germany, on August 22, 1903, aged 70 years.

WILLIAM CARY BARRETT, M. D.

From the Illustrated Buffalo Express,
*Copyright*, 1903, by Geo. E. Matthews & Co.

Dr. Barrett was born in Monroe County, N. Y. He received his preliminary education at Kingsville Academy, Ohio, Carey Academy and Yates Academy, in that state, and for some years taught in various places. In 1864 he began the study of dentistry and took the degree of master of dental surgery in 1869. He practised in Warsaw until 1876, when he came to Buffalo. In 1880 he received the degree of M. D. from the University of Buffalo. The next year he gained the title of doctor of dental surgery from Pennsylvania.

Dr. Barrett, in 1885, began lecturing at the University of Buffalo, and in 1890 became professor of oral pathology. About the same time he was appointed to a professorship in the Chicago College of Dental Surgery of the Lake Forest University. Dr. Barrett retained his residence in Buffalo, however, and went to Chicago at stated intervals, to deliver his lectures and give the instruction belonging to his chair.

Upon the organisation of the dental department of the University of Buffalo in 1891, Dr. Barrett was elected dean of the faculty, and also held the professorship before referred to. He was also oral surgeon on the staff of the Buffalo General Hospital.

For some years Dr. Barrett edited the *Dental Practitioner*, and was the author of several monographs upon subjects connected with dental medicine.

Dr. Barrett was a member of the Erie County Medical Association, and of the American Medical Association. He was a member of the International Medical Congress (London, 1881); an honorary vice-president of the ninth congress (Washington, 1887); and of the tenth congress (Berlin, 1890). He was president of the State Dental Association in 1875 and 1876, and of the American Dental Association in 1886. He was an honorary member of many state, national and foreign professional societies.

Dr. Barrett had traveled much and studied in many hospitals. His pathological collection is very valuable.

Dr. Barrett was married in 1857 to Amelia Harris Ryerse, of Port Ryerse, Ont., who survives him. Mrs. Barrett was with her husband abroad and brought his remains home for interment. The faculty and alumni of the dental department of the University of Buffalo took appropriate action upon the receipt of the news of Dr. Barrett's death.

## Figures and Tables

**Figure f1:**